# GWAS in a Box: Statistical and Visual Analytics of Structured Associations via GenAMap

**DOI:** 10.1371/journal.pone.0097524

**Published:** 2014-06-06

**Authors:** Eric P. Xing, Ross E. Curtis, Georg Schoenherr, Seunghak Lee, Junming Yin, Kriti Puniyani, Wei Wu, Peter Kinnaird

**Affiliations:** 1 Machine Learning Department, Carnegie Mellon University, Pittsburgh, Pennsylvania, United States of America; 2 Joint Carnegie Mellon – University of Pittsburgh PhD Program in Computational Biology, Pittsburgh, Pennsylvania, United States of America; 3 Computer Science Department, Carnegie Mellon University, Pittsburgh, Pennsylvania, United States of America; 4 Lane Center for Computational Biology, Carnegie Mellon University, Pittsburgh, Pennsylvania, United States of America; 5 Human Computer Interaction Institute, Carnegie Mellon University, Pittsburgh, Pennsylvania, United States of America; 6 Language Technologies Institute, Carnegie Mellon University, Pittsburgh, Pennsylvania, United States of America; University of North Carolina, United States of America

## Abstract

With the continuous improvement in genotyping and molecular phenotyping technology and the decreasing typing cost, it is expected that in a few years, more and more clinical studies of complex diseases will recruit thousands of individuals for pan-omic genetic association analyses. Hence, there is a great need for algorithms and software tools that could scale up to the whole omic level, integrate different omic data, leverage rich structure information, and be easily accessible to non-technical users. We present GenAMap, an interactive analytics software platform that 1) automates the execution of principled machine learning methods that detect genome- and phenome-wide associations among genotypes, gene expression data, and clinical or other macroscopic traits, and 2) provides new visualization tools specifically designed to aid in the exploration of association mapping results. Algorithmically, GenAMap is based on a new paradigm for GWAS and PheWAS analysis, termed *structured association mapping*, which leverages various structures in the omic data. We demonstrate the function of GenAMap via a case study of the Brem and Kruglyak yeast dataset, and then apply it on a comprehensive eQTL analysis of the NIH heterogeneous stock mice dataset and report some interesting findings. GenAMap is available from http://sailing.cs.cmu.edu/genamap.

## Introduction

Advancements in high-throughput sequencing and molecular profiling technologies have made it both affordable and efficient to record DNA sequence variations over millions of genomic loci, to measure the abundance of transcripts of virtually all known coding sequences, and to collect a wide range of pathological phenotypes in statistically meaningful disease/control populations. This deluge of inter-related omic-scale data offers an unprecedented opportunity to investigate how organismal functions respond to molecular-level alterations and how network disorders affect phenotypic-level phenomena, which are fundamental to the understanding, diagnoses and treatments of complex diseases such as asthma, obesity, and cancer.

Many complex disease syndromes consist of a large number of highly related, rather than independent phenotypes. Differences between these syndromes involve the complex interplay of a large number of genomic variations that perturb the function of disease-related genes in the context of a regulatory network, rather than individually. Thus unraveling the causal genetic variations and understanding the mechanisms of consequent cell and tissue transformation requires an analysis that jointly considers the epistatic, pleiotropic, and plastic interactions of elements and modules within and between the genome (G), transcriptome (T), and phenome (P). For example, a plethora of evidence suggests that SNPs associated with complex traits are likely to be expression quantitative trait loci (eQTLs) [1], necessitating inclusion of gene expressions instead of (or in addition to) phenotypic traits as association responses. Indeed, gene expression data are now commonly used to integrate transcriptome information into association studies [2], [3], [4]. Successful integration of eQTL analysis into genome-wide association studies (GWAS) has led to the identification of new disease genes in humans and mice [5], [6], [7], [8]. However, until now, most popular approaches for genetic and molecular analysis of genetic associations were mainly based on classical statistical techniques, such as linkage analysis of selected markers [9], quantitative trait locus (QTL) mapping conducted over one phenotype and one marker genotype at a time and then corrected by multiple hypothesis testing [10], or indirect association analysis between markers and statistical representations of expression or clinical trait groups such as cluster means or principal components [11]. Such approaches yield crude, often either highly noisy or over-stringent signals of causal genetic variations. For example, a recent analysis showed that in adipose tissue, at a 5% FDR, expression levels of 17,080 (72.0%) genes were correlated with BMI [12]. Another analysis concluded that the identification of 164 genes in the expression intersection of four co-expression networks in human breast cancer “could not be expected by chance” [13]. In this paper, we present GenAMap, a statistically sound and computationally efficient machine learning platform and software system to address the theoretical and practical challenges involved in unraveling the interplay between disease-relevant elements in the genome, transcriptome, and phenome.

GenAMap is a software system built on principled machine learning algorithms that detect genome and phenome wide associations (GWA and PheWA) [14], [15], [16] among genotypes (SNPs), gene expression data, and clinical or other macroscopic traits for a given disease, taking into account the structural information within each of the three data types. GWA analysis is a popular strategy to determine how sequence variation affects the inheritance of phenotypic traits [14], [15]. Traditional GWA mapping usually screens for candidate SNPs using either an association test statistic between SNPs and clinical traits or case/control status [10], or via sparse regression to select causal SNPs [17]. These approaches have led to the successful identification of many so-called disease genes and susceptibility loci for a variety of diseases such as prostate cancer [18], diabetes [19], and Alzheimer’s disease [20]. However, the success of these studies (and other studies based on these approaches) is limited [21] because the discovered SNPs only explain a fraction of the disease heritability [14] or identify SNPs that do not affect protein sequence and thus have no known role that would affect the actual disease [2].

One of the major limitations of most traditional approaches that look for pairwise associations between SNPs and genes or multiple phenotypic traits is that they ignore the structural information within the genome, transcriptome, or phenome, such as linkage disequilibrium (LD) due to non-random recombination and modularity in co-expressed genes in common biological pathways. Such information holds the key to boosting the statistical power for GWA mapping because co-occurring weak signals, which independently can be mistaken as noise, become statistically significant when examined jointly in light of such prior structural information. The recent development of a new generation of GWAS algorithms, termed structured association mapping algorithms, utilizes structural and other prior information to discover genome-transcriptome-phenome associations [22], [23]. Initial studies have suggested that structured association mapping indeed leads to increased insight and greater statistical power in association studies. In this paper, we will systematically explore and integrate these new approaches.

Another barrier preventing more effective GWA mapping with modern statistical and machine learning technology is the lack of accessible software tools built on these new technologies such as structured association mapping. This problem has received even less attention from methodologists, and prevents widespread use of new GWA models and algorithms. For example, the power of structured association mapping comes with more sophisticated machine learning techniques that require greater specialization to run and interpret. Moreover, due to the data complexity, results from these algorithms become a sea of data that can be challenging to explore. The necessity to involve multi-omic scale data sets in modern GWA analysis can be operationally complex and confounding due to potentially overwhelming amount of patterns and signals and the lack of a handy software platform for analysis. In this paper, we address this issue with a highly integrated and general-purpose software system that allows knowledge about genome, transcriptome, or phenome structures to be leveraged algorithmically and visually to improve and enhance discovery in GWAS.

**Figure 1:**
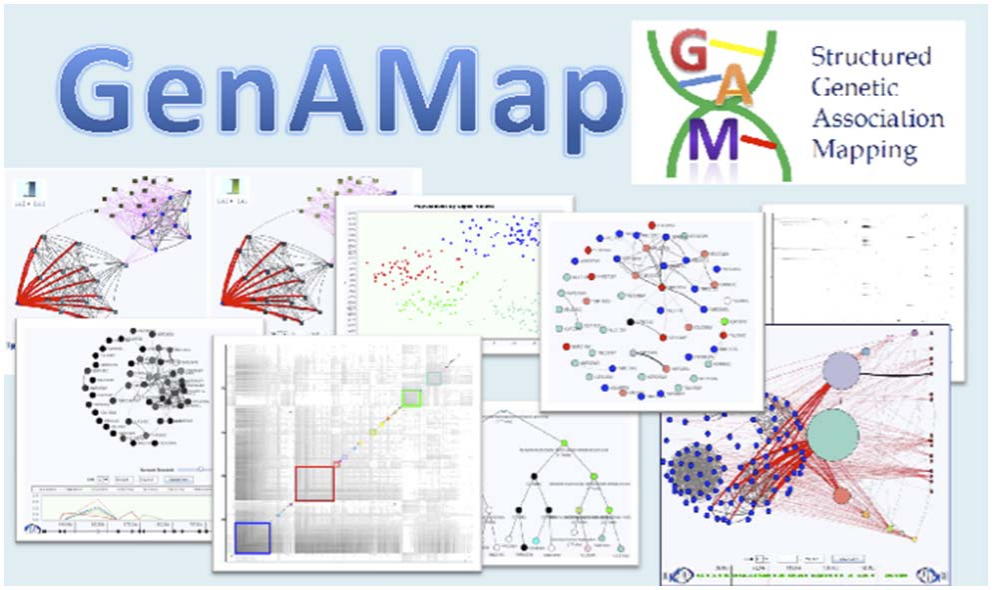
GenAMap is a visual analytics system for structured association mapping. Through the UI, users can explore the population and network structures of the data and determine which association analyses to run. Users can also take advantage of new, intuitive visualizations to explore the structures inherent in the data while simultaneously exploring the results from association analysis. All jobs are run on a remote cluster, and the results are displayed from the front end, linking out to external databases for further information and analysis.

The GenAMap system we present in this paper offers a new paradigm for GWAS and eQTL studies. GenAMap provides a rich collection of structured association mapping algorithms we have recently developed, along with classical GWA methods still widely in use, through a highly efficient and user-friendly human-computer interface. Through a graphical user interface, a user can invoke a combination of advanced algorithms and run them as a pipeline on complex datasets to map a set of co-expressed genes to a block of markers in the genome. More specifically, GenAMap focuses on building multivariate structured association models encompassing all three sources of omic data, relating sets of genotype markers (genome), to sets of gene expression measurements (transcriptome), and to sets of clinical trait measurements (phenome) in a joint genome-transcriptome-phenome association model. So far, there has been very little work analyzing these three resources under a unified framework to detect joint associations [19], [21], and no existing work considering the modules and structures in all three omics for association mapping. To our knowledge, GenAMap represents an initial foray into the development of a comprehensive statistical and visual analytics software system for structured association mapping that can 1) automate the execution of structured association mapping algorithms, and 2) provide new visualization tools specifically designed to aid in the exploration of association mapping results. A glimpse of the functions of GenAMap can be seen in Figure 1.

In the remainder of the paper, we present an overview of the statistical models and algorithms for structured association mapping built into GenAMap, followed by a discussion of the design and implementation of our system. Then we demonstrate GenAMap and the suite of new machine learning and visualization tools therein for GWAS and eQTL analysis through a case study using yeast data. Finally, we use GenAMap to analyze the NIH heterogeneous stock mice data [24]. By using structured association mapping and visualization in GenAMap, we find an eQTL hotspot on the mouse chromosome 14 that is associated with axon genes. We further investigate this eQTL hotspot and find specific genes associated with anxiety traits in mice. The three-way analysis using structured association mapping provides additional mechanistic insight into the SNP-to-clinical-trait association that has not been possible using other state-of-the art methods.

## Methods

### Structured Association Mapping

The main statistical paradigm upon which GenAMap is built is a new statistical formalism for GWA known as the sparse structured multivariate and multi-task regression (S^2^M^2^R). This emerging paradigm departs significantly from the traditional test-statistics-based [25] or PCA-based [26] methods (though they are also supported in our toolbox), which do not strongly leverage various structural information present in the genome, phenome, and transcriptome to improve the accuracy of identifying candidate causal variations in the DNA at a full-genome scale. S^2^M^2^R complements such inadequacy by exploiting a wide spectrum of omic structures available with the data as exemplified in this paper using a principled mathematical formalism that enjoys strong statistical guarantees and efficient computational algorithms, rather than using ad hoc heuristics of unknown asymptotic properties.

Specifically, the S^2^M^2^R formalism is built on the basic ideas behind lasso regression [27]. Lasso is advantageous in association mapping as it selects the most informative predictors (SNPs) for each response (gene expression or clinical trait) and eliminates false positives. Unlike single SNP analysis, the S^2^M^2^R formalism does not make the assumption that SNPs are independent and performs joint analysis considering all SNPs.

Let us begin with the following general definition of the association mapping problem. Let 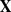 be an 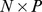 genotype matrix for 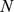 individuals and 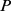 SNPs and let 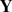 be an 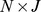 gene expression matrix where expression levels of 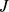 genes are measured for the same individuals, and let 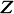 be an 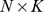 phenotype matrix where each row records 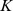 phenotypic traits of an individual. The basic lasso approach to finding associations between SNPs 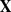 and traits 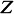 amounts to solving the optimization problem defined by the following equation:
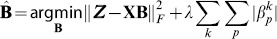
(1)where 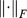 is the Frobenius norm of the matrix, the first term represents a penalty based on prediction error, and the second term is a sparsity-inducing L1 penalty that shrinks the strengths of irrelevant SNPs towards zero. In this scenario, 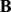 is a 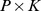 matrix, of which the non-zero elements represent the associations between SNPs and phenotypes.

In a more general setting, the first term in (1), known as the loss function, can be further elaborated to achieve various desirable effects, such as distinguishing continuous (e.g., a dose effect on traits) versus discrete (e.g., a binary effect on traits) responses [28] weakening assumption on noise and signal distribution [29], capturing non-linear effects [30], etc. In GenAMap, we follow common practice in the field and use a simple squared loss as shown above, but it is possible to update to more powerful forms by allowing the plug-in of alternative loss functions. The second term in Eq. (1), known as the shrinkage or penalty function, is where structural knowledge of the data can be systematically explored and exploited through the GenAMap. Below, we provide examples for the incorporation of the genome, phenome, and transcriptome structure, respectively, into the model.

#### Incorporation of genome structure

An important source of genome structural information is genome annotations that include known transcription factor binding sites, exon regions, transposable element locations, and conservation scores. These data can be considered as prior knowledge about SNPs that can be used to guide the search for association SNPs. For example, SNPs in highly conserved regions are more likely to be true association SNPs, as conserved regions are often functionally important. Taking advantage of genome annotation, Adaptive Multi-Task Lasso (AMTL) finds genome-transcriptome or genome-phenome associations [31]. AMTL defines different penalties to SNPs according to genome annotation (SNPs with small penalties are more likely to be selected), and simultaneously incorporates L1/L2 penalty to perform multi-task learning on correlated traits (to be discussed in the phenome structure section):
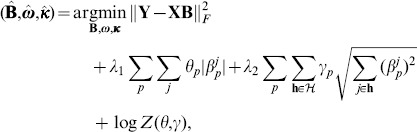
(2)where 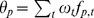, 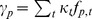 and
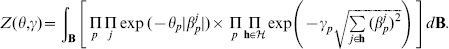
(3)

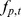
 represents the 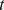-th feature score (e.g. conservation score) for the 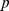-th SNP, and 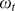 and 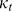 are the weights of the 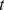-th feature for the two penalties, respectively. The model learns 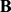, 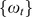 and 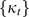 simultaneously. This is made possible by the term 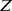, which acts as a regularizer on 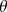 and 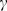 and hence on 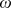 and 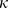. 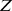 takes the form of a normalization term in a Bayesian probabilistic model for 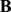. AMTL gives small penalty to SNPs with features desirable for association mapping and thus incorporates bias based on genome annotation.

The L1/L2 term employed above is also known as group lasso penalty [32], an extension of lasso, which can encourage simultaneous shrinkage of a set of SNPs known to be related from prior knowledge, and thereby enhance the statistical power of a GWA on high-dimensional genomic data based on a wide variety of structural knowledge beyond what is mentioned above. For example, in GenAMap, we also consider genome structures revealed through LD, biological pathways, and synthetic lethal gene-gene interactions. SNPs interrelated by such structures are likely to affect a gene expression value or a clinical trait in the same way, and the group lasso penalty captures such relatedness effectively and elegantly.

Another type of genome structural information is from the population structure. While many SNPs may be population-specific, some SNPs may have similar effects across populations. The multi-population group lasso (MPGL) is a sparse-regression method also built on the group lasso that allows associations to be discovered in different populations independently, while incorporating information across all populations [23].

#### Incorporation of transcriptome and phenome structure

Related clinical traits or gene expressions as revealed in a phenotypic network or a gene-expression clustering tend to be influenced by a common and small subset of SNPs. Biologically, this might be the case when a mutation in a genetic regulator affects expression levels of multiple genes in a common pathway. Such structural information present in the transcriptome or phenome introduces constraints on the output 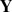 or 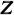 (instead of on 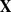 as seen in the genome case) of the S^2^M^2^R problem. Such structure bearing networks or clustering can be obtained using well-known machine learning techniques based on correlation or partial correlation, or from known gene-gene or protein-protein interactions that are experimentally validated.

The graph-guided fused lasso (GFlasso) [22] extends the lasso such that a network structure over the gene expressions is used to guide the discovery of associations. We define 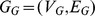 as a relevance graph where each node represents a gene in 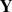 and each edge represents a weighted relationship between two nodes in the network graph. GFlasso is then described by the following optimization problem:
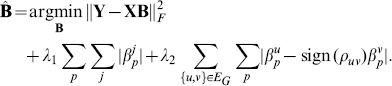
(4)In Eq. (4), 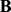 is a 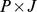 matrix representing genome-transcriptome associations. Here the second penalty term consists of a sum of the so-called total variation penalties defined over each edge of the network graph. This type of penalty encourages elements in 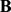, which correspond to the association strength of a SNP to a gene expression value, that are linked by the edge in the graph to attain similar magnitude, i.e., jointly zero or non-zeros. This strategy thereby enables structure information of the relationships between the gene expressions to influence the estimation of association signals. Similarly, we can create a network graph 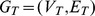 for clinical traits and substitute 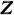 for 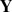 and 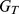 for 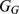 to find a 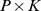 matrix representing genome-phenome associations.

A related approach to GFlasso is the TreeLasso [33]. TreeLasso builds a hierarchical clustering tree from the gene expression network (or clinical trait network) and uses the tree to represent the relationships between gene expressions or traits to guide the association discovery. Accordingly, a tree-penalty function built on a nested sum of L1/L2 norms over elements on different rows of 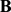 can be introduced to induce a hierarchical group sparsity pattern on 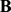.

#### Incorporation of genome-transcriptome or genome-phenome structure

So far, we have seen that genome structure and transcriptome/phenome structure can be incorporated into a regression model on the input or output side, rather than both. A natural extension of the two previous approaches is to incorporate both genome and transcriptome/phenome structures into a single model and exploit the synergistic effects of both structures. Suppose that groups of SNPs and groups of gene expressions/traits are determined a priori by a genome structure and gene expression or trait network. Then structured input/output multi-task regression [30] solves the following problem considering both structures simultaneously:
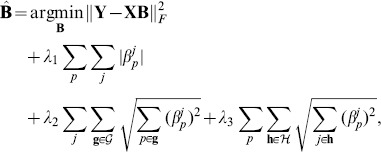
(5)where 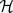 is the set of groups of gene expressions/phenotypic traits, and 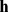 is a member of the set 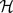. Under this model, coefficients for a set of correlated SNPs (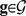) and a set of correlated gene expressions or phenotypic traits (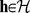) tend to be zero together due to the L1/L2 penalties, and at the same time, individual coefficients can be zero due to the L1 penalty. This model takes advantage of both genome structure and transcriptome/phenome structure in a sense that it can set coefficients to zero guided by both SNP groups and gene expression or phenotypic trait groups.

#### Joint three-way analysis

Finally, we consider a structured association mapping approach that uses combined genome, transcriptome, and phenome data to perform a joint three-way association analysis, GFlasso-gGFlasso [34]. This is done through a two-stage process. First, we find genome-transcriptome associations using GFlasso as just described. Next, we find transcriptome-phenome associations using the graph-Graph-guided fused lasso (gGFlasso):
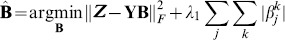
(6a)

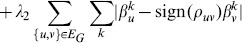
(6b)

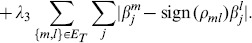
(6c)


In gGFlasso, we add a second fusion penalty to the GFlasso framework to encourage related genes in the gene-expression network to influence related traits in the trait network. This model assumes that genes in the same pathways might have similar effects on multiple related traits.

#### Optimization algorithms

For all aforementioned models, we can represent the optimization problems in the form of:
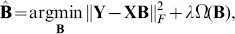
(7)where 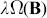 is non-differentiable and often non-separable convex penalty. Classical convex optimization techniques such as quadratic programming [35] and the subgradient descent method [35] do not scale well to large problems with hundreds of thousands of SNPs and traits. The coordinate descent method [36] is very efficient for lasso problems, however, it is not applicable to our models because the penalty of GFlasso and the TreeLasso is non-separable, and in such a case, coordinate descent does not guarantee convergence [37].

The key idea behind our optimization is as follows. We transform a non-smooth/non-separable form of penalties into a smooth/separable form which is easy to deal with. (Transformation can be done at the cost of approximation such as a proximal smoother [38], or adding additional constraints such as dual decomposition [39].) We then solve Eq. (7) using an efficient optimization technique such as a coordinate descent [36] or FISTA [40]. For example, in case of GFlasso/GFlasso-gGFlasso, we transform the non-smooth form of penalty into a smooth form with additional variables in constraints [22], [34]. Then the coordinate descent method is used to optimize the smooth objective, and estimate additional variables. In case of the TreeLasso, we adopt the smoothing proximal gradient method [38]. This method first makes the non-separable penalty separable by converting it into its dual form, and then makes it smooth by using a general smoothing technique [41]. After the transformation, the FISTA [40] method is employed to optimize the separable and smooth objective function. For AMTL, we deal with the non-separable penalty by checking group sparsity and individual sparsity consecutively. This technique can be applied due to special form of the penalty [42]. We alternatively estimate 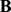 using a coordinate descent method, and estimate the weights of SNP features using a gradient descent method. The penalty of MPGL is non-smooth and separable, and thus we can optimize MPGL objective using an efficient method for standard group lasso [43] that solves the dual form of the original problem.

#### Estimation of significance

An attractive property of the traditional methods particularly adored in the medical genetics community is that they offer a p-value that reflects the significance of the findings. Quantifying statistical significance of the results from S^2^M^2^R remains an open problem that is actively studied in the statistics community [44], but we argue that in the nowadays ultra high-dimensional GWAS era (i.e., millions of SNPs and tens of thousands of traits) where statistical significance scores computed by classical means become less meaningful and usually inaccurate, S^2^M^2^R offers many unique advantages by allowing the abundance of biological prior knowledge on the data to be easily and directly incorporated (rather than used in pre-screening data or post-processing results) in the detection of association signals with enhanced signal to noise ratio. Recently, several methods have been proposed to compute p-values for high-dimensional regression [44], [45], and we can further advance these techniques to compute p-values for S^2^M^2^R. For example, the ‘screen and clean’ procedure [46] enables p-value computation by randomly dividing samples into two sets. However, this method may generate unstable p-values due to random splitting procedure. The ‘multi-split’ method aggregates p-values from multiple data splitting, and was shown to be more robust to noise induced by random permutation [44]. In the current version of GenAMap, the p-value computation is not included, but it will be incorporated in our next release.

### The GenAMap Software System

In addition to the needs for high-performance structured association tools for GWAS, there remains a huge gap between the active invention of new models and algorithms from the methodological community and the adoption of these new methods in the genetics and medical community. Two key obstacles hinder these advances from being widely accepted in practice: the expertise required to run structured association mapping algorithms, often from command-line implementations; and the lack of a convenient human-computer interface to explore the results after the algorithms complete.

**Figure 2:**
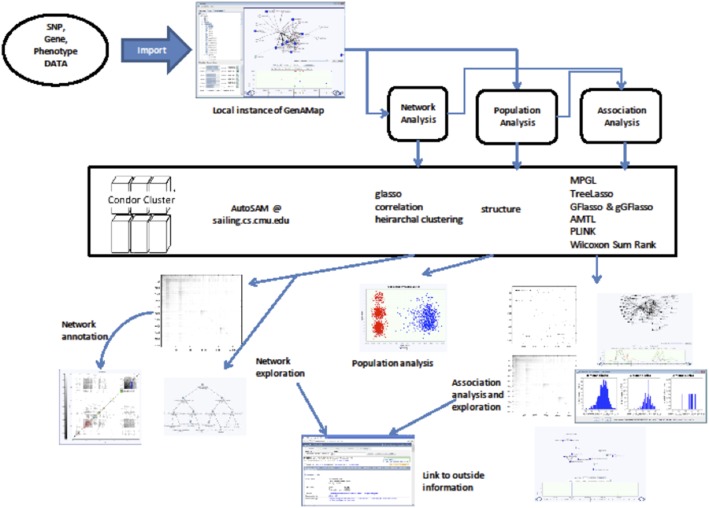
GenAMap Overview. GenAMap is run locally as a desktop application. It communicates directly with our cluster through Auto-SAM, an automatic system for running structured association algorithms. GenAMap executes all tasks, returning to the user a set of visualizations to explore and analyze the results to find interesting signals in the data.

We present a portable software suite called GenAMap that packages all the S^2^M^2^R GWA mapping tools we have developed so far, and new tools to come in the future, as well as traditional association methods such as PLINK’s WALD test [10] into a highly standardized, user interface (UI)-enabled platform that supports flexible data/result management, automatic task distribution in a multicore parallel computing environment, powerful visualization and interactive analysis, and a rich suite of graphical result formats. An overview of GenAMap can be seen in Figure 2. GenAMap is run locally as a downloadable software. It interfaces directly with an online computer cluster to run structured association mapping jobs and to collect and interpret the results. It allows users to import their own data for visualization and analysis. Below, we detail the two key components of GenAMap: automation of the S^2^M^2^R and selected GWA algorithms, and visualization tools needed to explore the results.

#### Automation

Most, if not all, mathematically sophisticated structured association mapping algorithms are generally made available as crude, command-line implementations (if they are made available at all). Thus, for a geneticist to use these algorithms, one must download a rough implementation of the algorithm and customize the code to fit his/her study. In contrast to this unfortunate state-of-the-art practice, as part of the GenAMap system, we incorporate an end-user-friendly strategy for the deployment of new statistical and machine learning algorithms to increase their accessibility for geneticists and biologists.

GenAMap runs structured association mapping algorithms through an automatic backend processing system called Auto-SAM [47]; additionally it also supports a variety of functions including structure-generating algorithms and other classical association algorithms (listed in Table 1). In contrast to the general strategy of posting a raw implementation on the web, we systematically develop and deploy each algorithm so that it will automatically run in a distributed parallel-computing environment. Thus, little technical specialization is required for a genetics analyst to pick up GenAMap and run the algorithms.

**Table 1:** Algorithms available to run in GenAMap.

Type	Algorithm
Structured Association Mapping	GFLasso [22]
	MPGL [23]
	TreeLasso [33]
	AMTL [31]
	gGFlasso [34]
Pairwise Association	Wald Test [10]
	Lasso [36]
	Association by population [84]
Network Generation	Correlation
	Glasso [49]
	Scale-free network [69]
Tree Generation	Hierarchical clustering
Population Assignment	Structure [88]
Gene network analysis	Gene module discovery [64]
	Hierarchical clustering

To generate structure, Auto-SAM provides algorithms to build networks and cluster trees, and find population structure. GenAMap runs baseline association methods through Auto-SAM including PLINK’s chi-square and Wald tests [10]. Most notably, GenAMap automates five structured association mapping algorithms: GFlasso, TreeLasso, AMTL, MPGL, and gGFlasso. Analysts can also load their own structures and results into GenAMap, bypassing Auto-SAM and using GenAMap’s visualizations to analyze the association results from any algorithm.

Our approach of distributing structured association mapping algorithms through Auto-SAM has several advantages over other distribution methods such as CRAN-R [48] (for examples see glasso [49] or bioconductor [50]): 1) By running algorithms on a distributed system with access to a cluster-computing system, Auto-SAM is able to handle much larger datasets and run algorithms in parallel; 2) through the use of a database, analyses are made available to entire teams of analysts; 3) the integration of Auto-SAM with GenAMap provides state-of-the-art visual analytic tools that enable the analyst to explore and analyze the data and results, including links to external databases and integration with gene ontology (GO) resources.

#### Visualization

Another key challenge in GWAS is the creation of a rich and unified visualization framework for a diverse spectrum of analytical and graphical needs. The vast amount of input and output to the structured association mapping algorithms and the sparseness of useful output classically suggests that a visualization strategy will aid analysts in the exploration of these data to identify the links between SNPs, gene expressions data, and clinical traits to eventually produce new treatments for disease.

Our design of the visualization scheme for analyzing GWA is built on the following insights. Once an analyst has run structured association mapping algorithms, the focus of the investigation becomes more exploratory than query driven [51]. Information visualization, “the use of computer-supported, interactive visual representations of data to amplify cognition”, as a field, touts its strengths as generating exploration-based insights, explanatory and persuasive interaction, and aesthetic representations [52]. Visualization techniques, therefore, excel when providing an explanation of the overall structure of the data or guiding analysts to weak or unexpected patterns most easily recognized by humans [52]. These are critical requirements for association analysis.

Indeed, the success of visualization strategies has emerged already in many areas of biology. For example, Cytoscape [53] has become an extremely popular application for visualizing biological networks and exploring relationships between genes. In other domains, the recent development of ABySS-Explorer [54] has shown that visualization can enhance the analysis of complex biological tasks like genome assembly through a visual representation of the contigs. Another recent approach to visualization in biology, MulteeSum, demonstrated the potential for visualization to aid in the identification of spatial and temporal patterns in gene expression data [55]. For simple GWA with one trait, excellent visualization tools have been built to explore LD, strength of associations, and surrounding genes in the association results [56], [57]. In GenAMap, we use multiple coordinated views to enable analysts to explore the structures of the genome, transcriptome, and phenome simultaneously when performing association analysis. In our experience, in a structured association study, researchers first need to get an overall picture of the patterns of associations in the data, and then they need to focus their attention on specific, important signals in the data. This immediately suggests a visualization strategy following Shneiderman’s well-known mantra: overview first, zoom and filter, details on demand [58]. As we will show, this mantra provides an excellent strategy for the development of the visualizations that guide discovery in association studies.

The GenAMap front-end interface is implemented in Java SE. To facilitate the rapid development of high-quality visualizations, we integrate and customize open-source visualization Java toolkits into GenAMap, including JUNG [59] for network visualization, JHeatChart [60] for an overview of network and association analyses, and JFreeChart [61] for detailed histograms and other scatter plots. The UI front-end of GenAMap communicates with Auto-SAM, the automatic processing system, through an Apache web-interface to our computing cluster. All data used by GenAMap are stored via MySQL. Auto-SAM executes algorithms from whatever technology they come from, including Java, C++, R [48], and MATLAB. Algorithms are parallelized and run using Condor [62]. Auto-SAM itself is written in Java.

In the following sub-section, we will demonstrate the capabilities of GneAMap using a yeast data set. While we only have space to discuss a single data set, users of GenAMap are free to upload their own data into the software, which can take any of the forms discussed in this paper. They may upload raw data in the form of SNP data, gene expression data or phenotype data. They may also upload the results of externally conducted analyses. This may take the form of a gene networks or of SNP-gene associations as discussed in the following section but also, for example, of a population structure as discussed later in this paper. A list of the major data set types available in GenAMap is shown in Table 2. All data sets, whether internally generated or externally generated and uploaded, can be viewed with the visualization tools as well as used for follow-up algorithmic analysis in GenAMap. Furthermore, GenAMap is fully applicable to data from a wide spectrum of sources including human subject data.

**Table 2:** Major data set types available to import/create via an algorithm in GenAMap.

Name	Description	Importing	Creation
Marker Data	SNP values of samples	yes	no
Trait Data	Gene expression or phenotypic trait data	yes	no
Trait Network	Network representing relatedness between traits (e.g. genes, phenotypes)	yes	yes
Association Data	Pairs of SNPs and traits; Result of association analysis	yes	yes
3-way Association Data	Associations between SNPs, genes and phenotypic traits	no	yes
Population Structure	Assignment of individual samples to populations	yes	yes
SNP features	Quantitative SNP information used as input to AMTL algorithm	yes	no
Trait Tree	Tree structure over traits indicating relatedness	yes	yes
Trait Clustering	Linear ordering of traits indicating relatedness	yes	yes
Trait Module	Group of highly related traits	no	yes

### Illustration: Analyzing Yeast Gene Networks and eQTLs

We now demonstrate GenAMap through an illustrative analysis. We use the Saccharomyces cerevisiae dataset from [63]. This dataset was generated by crossing a laboratory strain (BY4716) of yeast with a wild-type vineyard strain (RM11-1a) to create 112 progeny yeast strains. Each of the 114 strains were genotyped by microarray for 1260 unique SNP markers. Hence, if a true causal SNP was not genotyped, we can only hope to detect a proxy SNP that is correlated to the causal SNP. Gene expression data was also collected from each strain for over 6000 genes. Because this dataset has been extensively studied [64], [63], [65], [66], it serves as an excellent benchmark dataset to highlight the capabilities of structured association mapping and GenAMap in a scenario where plenty is already known about the associations in the data for verification, but additional patterns could still be uncovered due to systematic use of structural knowledge about the data via GenAMap. After preprocessing the gene expression data, we used 5637 gene expression measurements for each yeast strain. The data collection and preprocessing steps were completed independently outside of the GenAMap software system.

#### Importing SNP data and preparing for AMTL analysis

**Figure 3:**
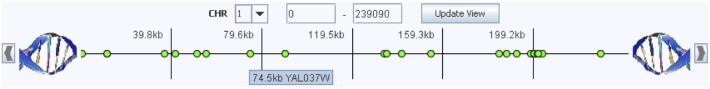
GenAMaps genome browser. GenAMap provides a simple genome browser that allows analysts to explore the mutation marker data that they load into GenAMap. SNPs are represented by green circles across the genome. Analysts can use these SNPs to directly link to external databases, such as SGD or dbSNP. SNP labels are displayed as the analyst hovers over the SNPs.

We import the SNP data as a tab-delimited file into GenAMap using the import wizard. When the import finishes, we can explore the data using GenAMap’s genome browser (Figure 3). It is a simple chromosome-by-chromosome browser that displays each SNP as a green circle, and can be used to check the distribution of SNPs on each chromosome and to directly link to the Saccharomyces Genome Database (SGD) [67] or dbSNP [68] for more information about the SNPs.

To prepare for in-depth analyses, we download and standardize twelve features from the SGD for each SNP and add these features to the dataset in GenAMap. These features include eleven discrete variables describing the locations of the SNPs (intron region, binding site, exon, etc.) and one continuous variable (conservation score) [31]. These features can be used as prior knowledge in AMTL in such a way that a priori belief on SNP associations is determined by weighted combination of the SNP features. Thus SNPs in annotated genomic regions (e.g. exon) are more likely to be selected than SNPs in unknown regions. As we browse through the SNPs, we can request to see the values of these features for a particular SNP by right-clicking on a selected SNP (or many selected SNPs) in the genome browser.

A problem may arise in case a causal SNP was not genotyped and we hope to detect it through a proxy SNP. If the features of these SNPs were highly dissimilar, using them in our analysis might hinder discovery. In order to have any hope to discover a causal SNP through a proxy SNP, they must be highly correlated. This implies, with high probability, that they both lie on the same LD structure. This, in turn, means that their features are likely to be similar. We believe that this similarity justifies the use of proxy SNP features in the AMTL regression model.

#### Network inference and exploration of expression traits

From a UI menu, one can easily load the gene expression data into GenAMap using the import wizard. Once uploaded, GenAMap provides several options for the analyst to automatically build a gene network, including the soft-thresholding method for scale-free network [69], pairwise correlation for correlation network, or glasso for Markov network [49]. An overall picture of the gene interactions is provided to help understand the network structure. GenAMap supports this type of analysis through the discovery of gene modules within the network. A gene module in GenAMap is a group of genes that cluster together. GenAMap analyzes these modules automatically to find GO functional enrichment and eQTL enrichment (when available).

**Figure 4:**
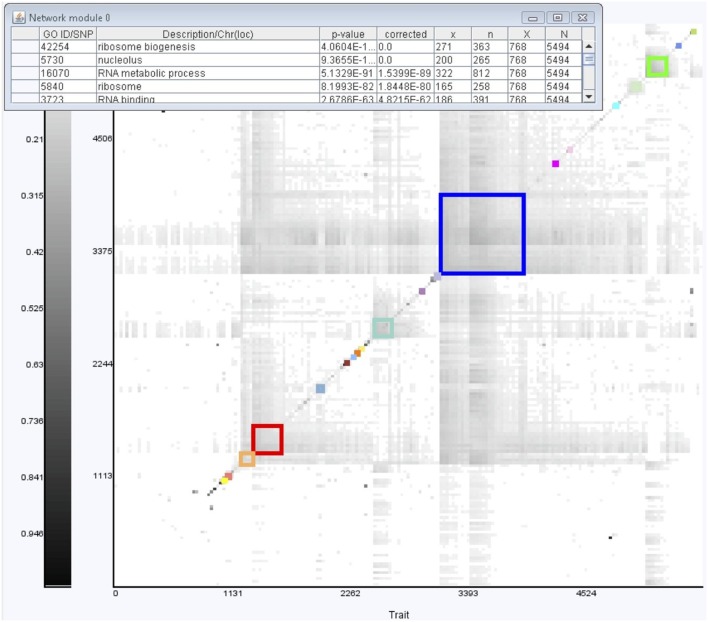
GenAMap trait overview exploration. GenAMap provides an overview of gene and phenotypic trait networks to aid analysts in their exploration of the networks. Here, we present a genetic network generated from the yeast data. The network has been further organized by hierarchical clustering, and twenty highly connected gene modules have been automatically identified by GenAMap (outlined in color). As the analyst clicks in these different modules, an information display appears to report the GO and eQTL enrichment of the genes that belong to the particular module.

We use GenAMap to run hierarchical clustering to cluster highly connected genes in the network and identify top twenty gene modules. This can be achieved on a parallel computing cluster by using an algorithm previously described in [64], which is supported by GenAMap. Simultaneously, GenAMap calculates the GO enrichment (using BiNGO [70]) for each discovered module. The GenAMap visualization tools allow us to interactively explore this gene network and the modules. Figure 4 shows a screen-shot of an overview visualization of the gene network. It is presented as a heat map, where darker pixels represent a weighted relationship between genes. The genes in the heat map have been clustered, and 20 identified modules are outlined in color. As we select different gene modules in the network, GenAMap displays the module’s GO enrichment results (and eQTL enrichment when available). In the yeast dataset we analyzed, we find that the modules are significantly enriched for certain GO categories, consistent with previous reports [64]. For example, we see that the blue module in Figure 4 is enriched for the GO category ribosome biogenesis.

As we mentioned in the design section, all of GenAMap’s visualization tools are developed to give an overview first, provide tools to zoom and filter, and then link to details on demand. The network view follows this pattern. Once we have an overview picture of the gene network, we use GenAMap to drill down into the data to explore interesting sub-networks. We provide one simple example of this type of top-down exploration.

**Figure 5:**
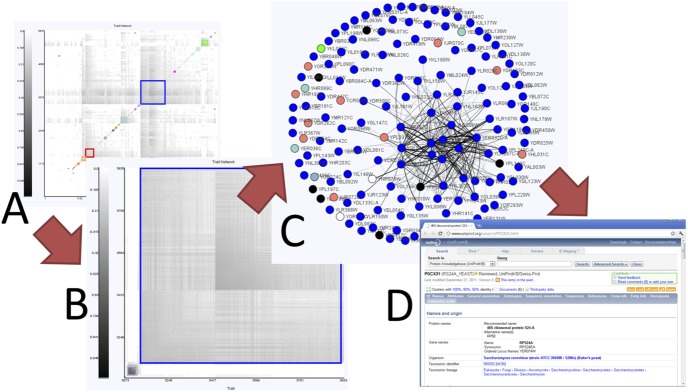
Using GenAMap to explore genetic networks. We demonstrate using GenAMap visualizations to explore a genetic network. A) From the overview of the network, the analyst can see the different gene modules in the network. B) The analyst zooms into a module of interest in the network. C) The analyst switches to a node-edge representation of this sub-network and adjusts the edge threshold, layout, and labels. D) The analyst uses GenAMap to link directly to external data sources for more information.

From the network overview, we observe that the largest sub-network is the blue sub-network, made up of 788 genes. This sub-network is enriched for many GO categories including “ribosome biogenesis” (p-value = 4.06e-169). To explore this sub-network further, we manually zoom into this region of the heat map display. GenAMap displays gene-expression and trait networks at a series of resolutions, so as we zoom into this region of the network we see the finer detail of the gene-gene relationships (Figure 5). We select the most highly connected part of the network and switch to the JUNG view, which displays sub-networks of up to 200 traits/gene-expressions in a ball and stick representation. We summarize this process in Figure 5.

In the JUNG view, genes are represented as circle nodes, and relationships between genes in the network are represented as weighted lines. Thicker lines imply a strong weight/degree of connection/correlation between genes. There are several different layouts available in this view, which include a simple circle layout and the KK-layout [71] shown in Figure 5C. Now that we have zoomed into this region, we use GenAMap to get details about these genes. We perform a GO enrichment analysis which finds that the selected genes are enriched for the GO category ribosome (p-value = 4.89e-169). We adjust the edge threshold manually to remove edges with lower weights. Because the top-connected genes in this network may be important players in the sub-network, we right-click on the labels of these genes to link directly to Google search and to UniProt [72]. These details on demand help us understand functions of the genes in this sub-network; for example *RPS24A*, a ribosomal protein from chromosome V, is the one with the most connections in the studied network.

#### Finding eQTLs through S^2^M^2^R

**Figure 6:**
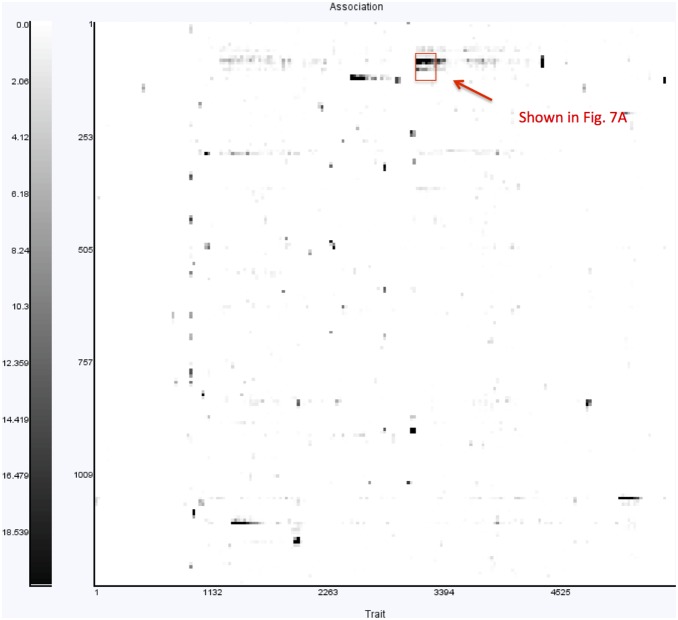
GenAMap overview of association results. GenAMap provides a heat chart visualization to explore the results from an eQTL association analysis. SNPs are plotted along the x-axis and genes are clustered along the y-axis. This view allows the analyst to explore the overview of the results. For example, in these results from running TreeLasso on the yeast data, many SNPs are associated with all the genes in a gene module, and some gene modules are associated with many different SNPs in different genomic locations.

Given the high modularity of the gene network, we decide to run the TreeLasso [33] to find SNPs associated with the genes that are inter-correlated under the cluster hierarchy produced in the earlier step. In Figure 6, we present an overview of the results from running the TreeLasso automatically in GenAMap. This view shows a heat map where SNPs are plotted along the y-axis and the genes plotted along the x-axis. The discovered associations between SNPs and genes are represented by the dark pixels in the plot, whereas white pixels represent no associations.

**Figure 7:**
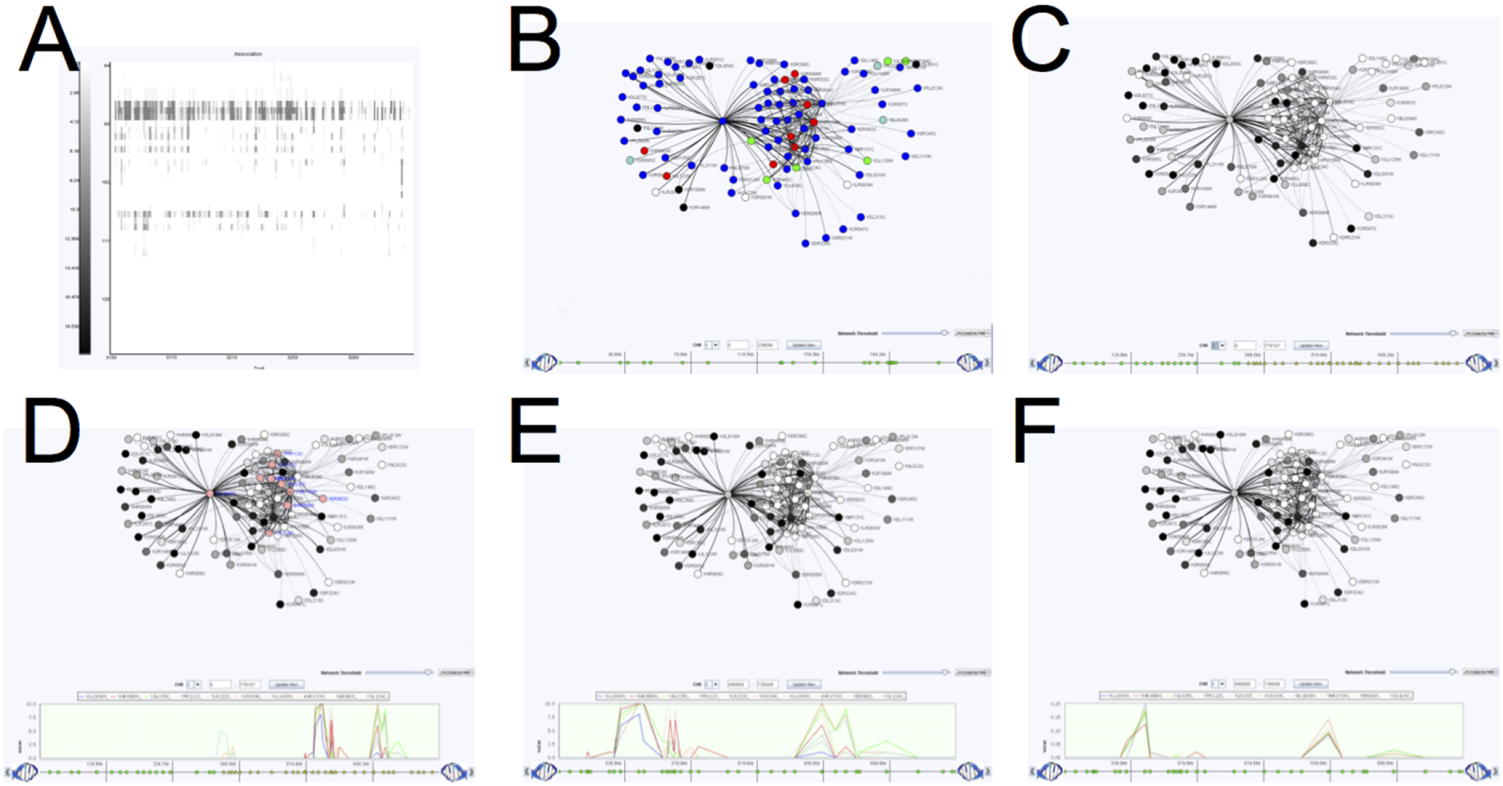
Using GenAMap to find eQTLs in yeast data. GenAMap provides many tools for analysts to explore association results while using the structure of the data to guide the discovery of associations. We demonstrate some of these tools. A) The analyst can zoom into certain regions to see finer detail of the SNP-phenotypic trait associations. This panel is a zoomed-in region from Figure 6. B) The analyst switches to the JUNG view to explore the genes associated with the region and perform a GO enrichment test. C) The analyst colors the genes by strength of association to the genomic region. D) The analyst selects up to ten interesting genes (salmon colored) and views the Manhattan plot of associations from these genes across the genome. E) The analyst zooms into interesting regions in the genome view. F) The analyst can switch between association tests for further insight into the associations.

From the results shown in Figure 6, we observe that many SNPs are associated with clusters of genes, meaning that the associations follow the modular structure of the data. We also observe that many of the gene sub-networks are associated with more than one SNP, suggesting some kind of interaction between the SNPs to regulate or affect the gene expression of the modules. We zoom into the heat map to see the finer structure of the associations in the largest cluster (Figure 7A), and notice that there are ten SNPs in the same genomic region that are associated with these genes. To explore these associations, we select the 131 genes in the cluster with strong associations and switch to the JUNG view.

Once in the JUNG view, we perform a GO enrichment test to see if the selected genes have common functions. Indeed, the genes are enriched for the GO annotations “nucleolus” (p-value = 2.09e-107), “ribosome biogenesis” (p-value = 2.62e-99) and “RNA metabolic process” (p-value = 7.08e-66). Figure 7B shows these genes color-coded by GO category, e.g., all genes with the GO annotation “nucleus” are shown in blue. These results suggest that the selected genes are involved in ribosome biogenesis in the nucleolus.

From the earlier exploration we performed with the functional gene modules and the gene interaction network, we know that these functionally coherent genes have strong associations to at least ten SNPs on chromosome II (Figure 7A). We select half of chromosome II and color the genes by degrees of associations to the selected SNPs (Figure 7C), e.g., genes with strong associations to these SNPs are shown in white, with weaker associations shown in gray. In this view, the SNPs being considered are shown as yellow triangles at the bottom of each panel for our reference. We find that expressions of most of the genes in this sub-network are associated to the same region on chromosome II. To further explore these associations, we select ten genes in the module (highlighted in salmon in Figure 7D) and view the association strengths of these genes across chromosome II using the Manhattan plot (Figure 7D, below). Then we zoom into the region with the strongest associations, and notice that these genes are associated with many SNPs (Figure 7E).

Since we have already added feature data to the SNPs, we run AMTL to find the SNPs most likely to be associated with the genes in this module. Unlike the TreeLasso, AMTL takes into account SNP features instead of genetic structure, and selects SNP-gene expression associations with bias toward SNPs having genomic annotations (e.g. SNPs on exon regions); this allows us to filter out many false positive SNPs with weak associations in unknown regions. Once the AMTL analysis is complete, GenAMap allows us to switch between the TreeLasso- and the AMTL results (Figure 7E and 7F), and even to combine the results from both methods if desired. Indeed, compared to the TreeLasso, AMTL finds associations to far fewer SNPs on chromosome II for the selected ten genes. To further explore the two SNPs on chromosome II as revealed by AMTL, we look into their information on the SGD, which GenAMap links to, and find that one of the SNPs is in *RPB5*, a component of RNA polymerases, and the other SNP is in *PYC2*.

In summary, in this demonstration using the yeast data, we have shown how GenAMap enables an analyst to inspect a gene expression network to find modules of interest and then drill down further to get details about them. We have also demonstrated how we can use GenAMap to explore association results and gene modules under the regulation of eQTL hotspots. Furthermore, we have shown how GenAMap allows analysts to compare the results from different structured association mapping methods to better understand association signals.

### Obtaining GenAMap and Further Information

GenAMap is readily available for download from our Website [73]. The software comes complete with installation instructions and a range of video and text-based tutorials. If users have any further questions, they can contact the corresponding authors for more information.

## Results

In this section, we apply GenAMap to a case study on the NIH heterogeneous stock mice dataset [24] to further demonstrate the function of this software system, and also to report interesting biological findings from the study. Here, we highlight the structured association mapping methods available to run in GenAMap, and also describe the visualization tools available to explore the results from these analyses. Due to the space limit, some of the graphical illustrations that resemble others that were previously shown can be found in the online supporting information.

The mice dataset consists of more than 2500 mice that have been genotyped for 12,545 markers and phenotyped for more than 150 traits [74]. Additionally, gene expression profiling data were generated from the liver and the lung for 260 genotyped mice and also from the brain for 460 mice [75]. This dataset has been well studied for SNPs associated with the mouse phenotypic traits and gene expressions, and thus, it provides an excellent test bed to demonstrate the strength of structured association analyses.

To prepare the data for analysis, we preprocess the expression data from each tissue (liver, lung, and hippocampus) using lumi [76]. We retain all probes that have a significant signal (d
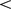
0.05) for at least 95% of the mice in each tissue, and keep the data from mice that have gene expression data across all three tissues. We impute missing phenotypic traits using k-nearest neighbor imputation [77], and exclude all phenotypes with missing values in more than 30% of the mice. In summary, our dataset contains 218 mice, each genotyped for 12545 SNPs, having measurements for 173 phenotypic traits, and having gene expression data from the liver (7102 probes), lung (9698 probes) and hippocampus (9733 probes).

Our analysis consists of four steps. First, we perform a network analysis of the gene expressions to uncover and visualize the correlation and cluster structures of the genes, which can in turn be used as prior information for the subsequent eQTL mapping. Then, we carry out a genome-wide eQTL analysis, using the S^2^M^2^R tool provided in GenAMap, to detect SNPs significantly associated with the gene expressions of interest. Next, we perform a three-way genome-transcriptome-phenome analysis in an attempt to establish a more complete picture of the statistical inter-dependencies between eQTLs, gene expressions, and the mouse phenotypic traits that suggest causal relationships between genetic variations and phenotypic diversity. Finally, we conduct a population analysis to explore the population structures in this dataset and their effects on associations.

### Network Analysis

After importing the data, we use GenAMap to construct gene expression networks for each tissue using the soft-thresholding method described in [69]. On each network, we performed hierarchical clustering and ran a dynamic programming algorithm [64] to find the top 20 connected gene modules. As a baseline, we used GenAMap to run PLINK [10] to find pairwise SNP-gene associations. GenAMap automatically considers all p-values less than 1e-3 to be significant, which, although naive in its approach, is a sufficient cutoff that allows us to get an overall idea of the associations in the dataset. For each of the 20 modules, GenAMap does eQTL and GO enrichment analyses. We use the GO slim annotation and the associations found by PLINK for this analysis. Figure S1 shows the annotated network generated from the gene expression data from the brain. The top connected modules GenAMap identifies are outlined in color.

Overall, we find that the gene networks in different tissues to be quite different. We identify a number of unique genes and unique edges between genes in the network of each tissue (see Table 3 for a summary of the comparison of the three networks). While many genes (e.g., 78% of the genes in the liver network) are shared between the three networks, few edges are common across the networks (e.g., only 14% of the edges in the liver network are common across all three tissues), suggesting the three tissue types have different regulatory patterns of expression.

**Table 3:** Comparison of gene networks across mouse tissues.

Tissue	#genes	% genes Sharedw/brain	% genes Sharedw/liver	% genesShared w/lung	% genesSharedacross tissues	#network edges	% edgesShared w/brain	% edgesShared w/liver	% edgesShared w/lung	% edges Sharedby all tissues
Brain	7960	100	59.6	78.1	57.6	170982	100	8.9	16.6	4.2
Liver	5879	80.8	100	86.8	78.0	48768	31.1	100	22.9	14.8
Lung	7968	78.1	64.0	100	57.6	105933	26.8	10.5	100	6.8

Also, we notice the gene expression networks in different tissues contain modules enriched with different GO groups and eQTLs. In the liver, we find nine gene expression sub-networks enriched for a GO groups including mitochondrion, catalytic activity, and generation of metabolites and energy (Table 4); whereas the hippocampus network has eight sub-networks enriched for GO groups, including ribosome, calcium ion binding, and transport. The eQTL enrichments for the modules are also different across the three tissues. Of note, we find five modules in the lung gene expression network significantly enriched with genes having association to the SNP *rs3023797*. No modules in the liver or brain are significantly enriched for association with this SNP. Interestingly, this SNP is located in the exon region of the gene *Ttf1*, which is a transcription termination factor of RNA polymerase I [78]. Ttf1 has previously been shown to play important regulatory roles in lung function and development in mice [79]. These results, therefore, suggest that mutations in Ttf1 affect expression patterns in the lung, but not in the other tissue types. Similarly, six gene expression sub-networks in the lung network also have an enrichment for association to chromosome 12, base pair 26000000, which is not seen in the other two tissues. This suggests that there is a second mutation that affects gene expression in the lung, but not in the liver or hippocampus.

**Table 4:** Gene modules with GO enrichment in the liver network.

Modulenumber	#genes inmodule	eQTL location	eQTLp-value	GO Category	GO p-value
1	446	11 (4877160)	1.47E-57	mitochondrion	3.80E-04
2	104	17 (61151939)	6.10E-07	catalytic activity	1.96E-04
4	201	14 (9353843)	7.42E-114	ion channel activity	2.02E-04
5	97	19 (20354841)	3.38E-31	mitochondrion	1.11E-13
8	89	17 (61151939)	1.81E-07	cytoplasm	3.73E-04
12	45	13 (56818025)	2.56E-10	regulation of gene expression epigenetic	5.59E-05
14	22	1 (76152963)	8.61E-07	generation of metabolites and energy	6.28E-04
15	34	19 (21138174)	4.18E-10	ER	7.08E-04
20	20	6 (42868138)	1.31E-11	nucleic acid binding	2.34E-04

### Structured Association Analysis of eQTLs

To leverage the network structure derived from the gene expression profiling data, which reveals potential correlational, regulatory, or even pleiotropic relationships among the gene expressions we employ the GFlasso algorithm [22] supported in GenAMap to identify eQTLs for each tissue type with enhanced statistical power over PLINK. GenAMap uses 10-fold cross-validation to find optimal values for the sparsity-inducing regularization coefficients 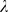 and 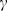 with a linear search strategy (documented at http://sailing.cs.cmu.edu/genamap). We download all results from GenAMap and collect all the identified SNP-gene associations. SNPs within 2 MB of each other and associated with the same gene are considered as the same association. In order to classify associations as *cis*- or *trans*-associations, we identify genomic locations of all genes [80].

Our results show significant differences in the eQTL patterns in the different tissues. In the liver, GFlasso identified six SNP-transcriptome associations, all of which are cis associations. (We define an association as a cis association when a SNP and its associated gene are located on the same chromosome and within 10MB of each other). The results from the lung were similarly sparse; GFlasso found 25 SNP-gene expression associations, one trans association and 24 cis associations. Overall, GFlasso identies two cis SNP-gene expression associations common across all three tissues (*Gps2* and *Psmb6*), one cis association common between liver and lung (*C4b*), and four cis associations common between lung and hippocampus (*Mrpl15*, *Hsd17b11*, *Rpl21*, *Hbb-b1*).

In contrast to the sparsity of the structured association results in liver and lung, GFlasso found many eQTLs using the hippocampus data. Specifically, we identified 467 SNP-gene expression associations for 103 SNPs and 268 genes, among which 138 are cis- and 329 are trans associations, 79 genes are associated with more than one SNP, and 6 SNPs are associated with more than 20 genes. Although the sparsity of the results for liver and lung is unexpected, our results are consistent with previous reports [75] that found that “trans-eQTLs are twice as common as cis-” in the brain, and that trans-eQTLs are much more common in the brain than in the other two tissues. It is the particular strength of GFlasso to detect associations between SNPs and multiple correlated traits as the algorithm combines the evidence for these by encouraging corresponding coefficients to have similar values through the fusion penalty. Since correlated traits are likely to be in different genomic locations, it is expected that GFlasso uncovers many trans associations. Because the results from the hippocampus are the most interesting, we will focus on these signals in the remainder of this section.

An overview of the SNP-gene expression association results for the hippocampus is presented in Figure S2. In particular, we notice one long horizontal line in the plot, suggesting the presence of an eQTL hotspot that regulates many genes in trans. We also notice the presence of other shorter horizontal lines, including some overlapping with some of the genes of the largest eQTL hotspot. Using GenAMap, we identify the SNP associated with these genes as *rs8244120* located on chromosome 14. We used GenAMap to find the SNP (*rs8244120*) in dbSNP and find that it is located in the exon coding region of two genes: *Tmem55b* and *Apex1*. *Apex1*, apurinic/apyrimidinic endonuclease 1, has been implicated in playing key roles in neuronal survival during ischemic brain injuries [81].

To better understand the genes associated with this genomic region, we use GenAMap to create a subset of all genes associated with *rs8244120*. GenAMap identifies 140 genes associated with *rs8244120* in the GFlasso results. Further GO analysis using GenAMap revealed that these associated genes are enriched with the genes involved in “cell projection” (p-value = 2.65e-5), which is defined as “A prolongation or process extending from the cell, e.g. a flagellum or axon” [82]. Indeed, many of the genes in this subset are annotated to GO categories indicating involvement in brain function, e.g. *Gas7*, *Nrp1*, *Stx1a* are annotated to the GO category neuron projection development. We select the 22 genes involving in “cell projection” and save them as a subset for further analysis. These 22 genes are enriched for many GO annotations including cell projection (p-value = 1.49e-27), neuron projection (p-value = 5.32e-17), axon (p-value = 3.30e-9) and dendrite (p-value = 2.65e-7).

**Figure 8:**
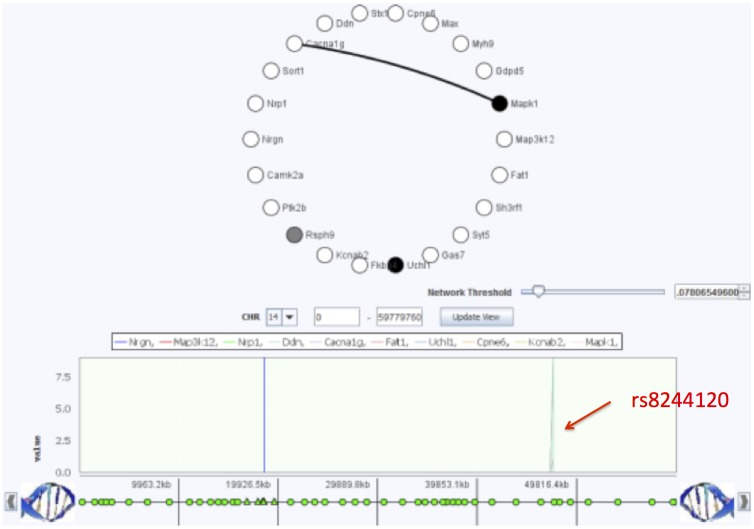
Association of axon genes to chromosome 14. We found that *rs8244120* on chromosome 14 was associated with 140 genes enriched for cell projection, implying function in neuronal axons. Here, we show 22 of the genes with the strongest associations in GenAMap’s node-link view, colored by the strength of association to *rs8244120*. White genes are strongly associated and black genes are weakly associated (gray is intermediate). We found that some of the genes were also associated with another SNP on chromosome 14 (shown) and some of the genes were associated with a SNP on chromosome 18 (not shown).

To further investigate the associations of the identified cell projection genes to the SNPs, we generate a Manhattan plot of the associations for the genes across GenAMap’s genome browser (Figure 8). We notice that all of the genes were associated with *rs8244120*, as expected, but that many genes had associations with other SNPs as well, e.g., two of the genes are also associated with *rs13482353* on chromosome 14, and three of the genes were associated with *rs3722205* on chromosome 18. We look into these two SNPs in more detail and find that 27 genes are associated with *rs13482353*, 25 of which are also associated with *rs8244120*. Likewise, 25 of the 27 genes associated with *rs3722205* are also associated with *rs8244120*. These results suggest that these SNPs may interact in some way to regulate gene expression in the mouse hippocampus.

Furthermore, we investigated an unrelated set of 41 genes associated with *rs1348069* on chromosome 10 in the GFlasso results. We find that these genes are enriched for several GO categories including extracellular ligand-gated ion channel activity (p-value = 2.20e-4), membrane depolarization (p-value = 2.54e-4), and synaptic transmission (p-value = 5.17e-4). *rs1348069* is in the intron region of *Slc5a8*, a gene involving in ion transport, suggesting that this SNP plays a role in ion-related activities by affecting these genes through altering the expression or function of *Slc5a8*.

### Three-way Genome-transcriptome-phenome Analysis

Given the results of our eQTL analysis, we decide to further run a three-way association analysis using GFlasso-gGFlasso to find multi-level associations from the clinical phenotypic traits to the genes in the brain and to the potential causal SNPs in the genome. We ignore all clinical traits that are marked as “Covariates,” since these are largely dates, experimenter ids, and other variables such as gender and litter. Overall, GFlasso-gGFlasso found 759 genome-transcriptome-phenome associations. These associations included 138 associations to the X chromosome, which we ignored due to possible gender effects. Thus, the results consist of 621 associations between 98 SNPs on 18 chromosomes to 156 gene expressions that are associated with 94 phenotypic traits.

We compare the GFlasso-gGFlasso results with those reported using a SNP-phenotypic trait association method [74]. We found nine matches where GFlasso-gGFlasso identified a SNP-gene expression-phenotypic trait association that matched the previously reported SNP-phenotypic trait associations (Table 5). While the previous analysis focused on SNP-to-phenotypic trait associations, the GFlasso-gGFlasso results suggest associated genes that help explain the previously discovered SNP-phenotypic trait associations. This is a particular strength of GFlasso-gGFlasso.

**Table 5:** GFlasso-gGFlasso results for the association analysis of the mice dataset.

SNP	Chromosome	Gene	Trait
*rs13459079*	4	*C1qb*	Alkaline phosphatase
*rs4226889*	7	*Nsmce1*	Weight at 6 weeks
*rs3718803*	11	*Pcdh20*	Aspartate Transaminase
*rs3023277*	11	*Psmb6*	Mean corpuscular haemglobin
*rs6326787*	11	*Gabrd*	Startle response
*rs6380524*	11	*Ube2g1*	Startle response
*rs4229111*	11	*Mpp3*	Startle response
*rs1348295*	17	*H2-T22*	CD4+/CD8+
*rs1348295*	17	*H2-T22*	%CD4+/CD3+
*rs1348295*	17	*H2-T22*	%C8+ cells

We show GFlasso-gGFlasso associations that match the previously identified associations by Valdar et al. [74].

**Figure 9:**
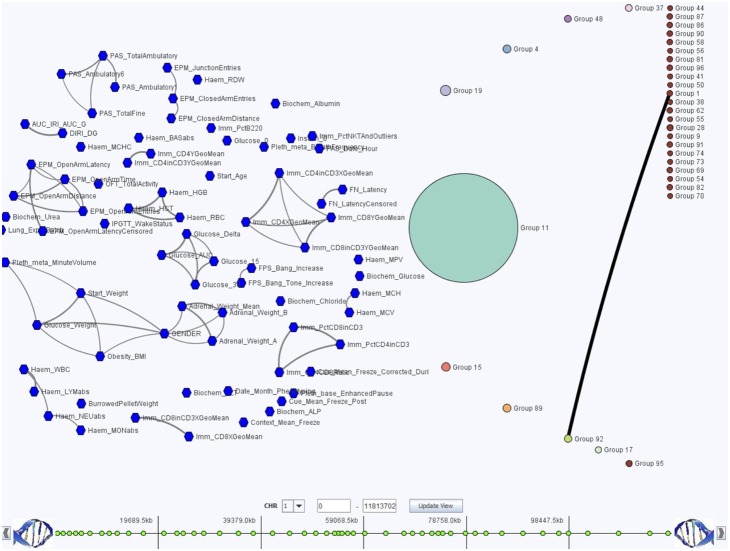
Overview of three way GFlasso-gGFlasso association analysis. We show the overview of the phenotypic trait-network and gene-network from GenAMap for the GFlasso-gGFlasso analysis; associations are not shown. In this visualization, circles represent groups of genes, associated to the same regions in the genome. Hexagons represent phenotypic traits. The edges between genes or between phenotypic traits represent the connections in the gene or trait network. In this data, we note that there are very few edges between gene groups. The largest gene group is the teal group, representing genes associated with the eQTL hotspot on chromosome 14. The phenotypic trait network consists of small sub-groups of related traits.

We use GenAMap to further explore the identified associations in the results. First, we consider the overall structure of the gene expressions and phenotypic trait data (Figure 9), noting that the largest gene group was associated with the eQTL hotspot on chromosome 14 as discovered in the previous section. To better understand the associations of these gene expressions to the phenotypic traits, we use GenAMap to zoom into these genes and the associated phenotypic traits and to filter out all other genes, phenotypic traits, and associations (Figure S3). After exploring the results, we are particularly interested in six genes that we find to be associated with sub-networks of anxiety traits (Elevated plus maze open arm time, distance, latency, etc.) due to the probable links between the brain and the traits. Figure S4 shows these traits, the correlations between traits (represented as gray lines between phenotypic traits), and the gene expression-phenotypic trait associations (pink lines between genes and phenotypic traits). We also find that the genes are associated with two regions on chromosome 14, which is consistent with previous findings showing two peaks on chromosome 14 associated with these phenotypic traits [74]. Furthermore, the results suggest potential mechanisms for these associations. For example, consider *Calb1*, a gene associated with the two eQTL hotspots and the anxiety traits. *Calb1* has been annotated to the axon, and *Calb1* knockout mice are known to show severe impairment in motor coordination [80]. Similarly, *Gabrd*, which is involved in ion transport [80], is also associated with one eQTL hotspot and the anxiety traits. *Gabrd* knockout mice have increased postpartum depression and anxiety. Thus, biological evidence supports the GFlasso-gGFlasso results suggesting mutations on chromosome 14 affect the expression levels of *Calb1* and *Gabrd* in the hippocampus, which in turn affect the anxiety traits.

We also consider other associations that GFlasso-gGFlasso uncovered. For example, the GFlasso-gGFlasso results find an association between chromosome 17 and immunology traits (CD4+/CD8+, CD4+/CD3+, and CD8+), which was also reported as a strong signal using the simple SNP-phenotypic trait association method [74]. To find out if GFlasso-gGFlasso can provide further mechanistic insight into this association, we use GenAMap to drill down to this association (Figure S5). We identify a gene group consisting of four genes that were associated with these immunology traits. One of the genes, *H2-T22*, is associated with all three correlated immunology traits. It is also associated with *rs13482952* on chromosome 17, which is 3.2MB away from the *H2-T22* coding region. Given that the resolution for this cross is about 2MB, this SNP likely affects expression of *H2-T22* in cis. In fact, this region on chromosome 17 is part of the mouse H2 region, the major histocompatibility complex (MHC). The H2 region is the mouse ortholog to the human HLA region and encodes genes involved in the mouse immune response [83]. The immunology traits associated with *H2-T22*: CD8+, CD4+, and CD3+, refer to the proteins on the surface of immune response cells that bind to the antigens on the surface of other cells in the organism. *H2-T22* is a membrane protein [80], and likely participates in this immune response pathway. As the immune response is common across all cell types, we would expect to find this association in all cells, including the brain tissues.

### Population Structure and its Effect on Associations

It is known that genetic admixing within complex populations can potentially confound association patterns. Therefore in this section we leverage another function of GenAMap to investigate such population structural effects.

**Figure 10:**
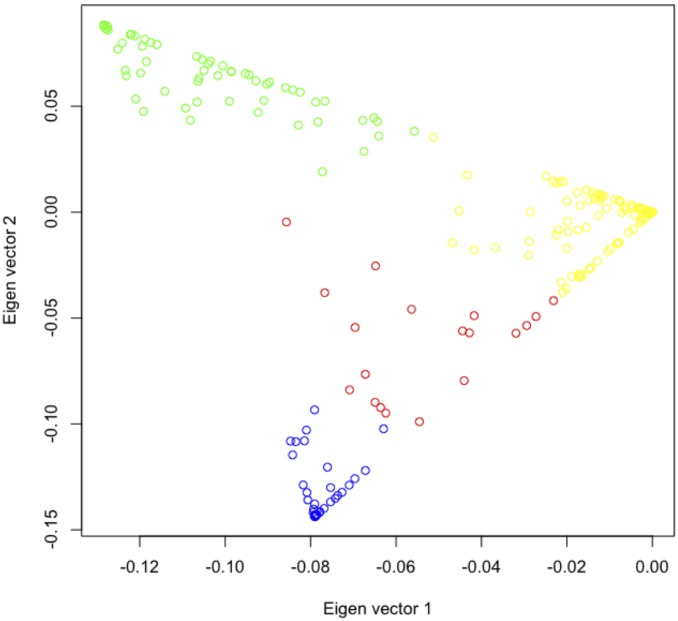
Analyzing population structure in GenAMap. GenAMap provides an interactive view for analysts to explore population structure. Population assignments are plotted by individual by Eigenvalue. The analyst can adjust the 2D plot to adjust between the first five Eigenvalues. Here, we present the results from a population analysis on the mouse data. The population label for each individual predicted by Structure is plotted according to the first two Eigenvalues. The plot shows clear separation between the populations.

While analyzing the Hippocampus SNP-gene expression associations, GenAMap finds a set of 22 genes highly correlated with SNP *rs8244120* on chromosome 14 (Figure 8). We ask whether the strength of these associations differs within heterogenous populations in the data. GenAMap uses Structure to automatically detect and stratify the population structure of the data. It then allows the user to explore the population structure by plotting the individuals by Eigenvalue. The analyst can explore 2D plots for the first five Eigenvalues to compare different numbers of populations in the data. In this dataset, we find that the populations separate into four distinct populations (Figure 10).

Given the strong separation between populations, we choose to perform multi-population group lasso (MPGL), which can analyze association differences across populations. In addition to MPGL, GenAMap also provides three other simple statistics to explore associations by population [84]. The three analyses are 1) the Wald (qualitative traits) or chi-squared likelihood (binary traits) test as implemented by PLINK [10], 2) a two-sided t-test on the phenotype distribution by genotype, and 3) a likelihood test [17]. Once this analysis completes, GenAMap provides visualization tools to explore the differences in association by population and by test; and an analysis tool to explore the similarities and differences in the results.

**Figure 11:**
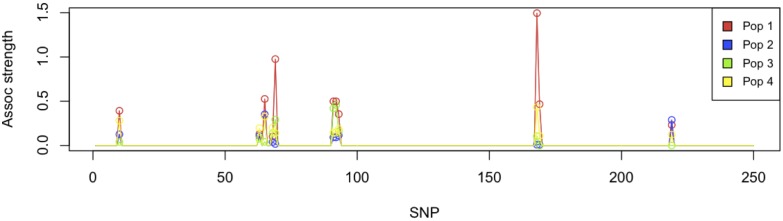
Interactive Manhattan plot for population data. GenAMap provides an interactive Manhattan plot for exploring associations in population data. Here we show the results of MPGL looking for genetic associations associated with the gene expression Mapk1 in the hippocampus gene expression data. The four colors represent the four populations in the data detected by Structure. The strength of the association in population 1 is significantly higher for all SNPs than in the other 3 populations.

In Figure 11, we present GenAMap’s visualizations built to explore association by population. Figure 11 shows a region on chromosome 14 where many associations were found to the gene expression Mapk1 in the hippocampus gene expression data, one of the 22 genes found to be associated with this region of the chromosome 14 (Figure 8). 19 out of the 22 genes were found to have a significant difference in the strength of the association across the 4 populations. In this interactive Manhattan plot, we can add and remove populations, as well as tests. For example, in Figure 11 we show the results from the MPGL for 4 populations, but we can also plot the PLINK and likelihood tests for these populations in the same figure.

**Figure 12:**
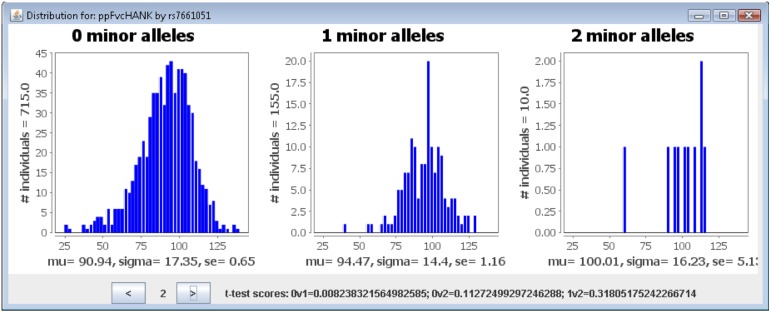
Frequency distribution of phenotypic trait by genotype. When exploring SNP-gene expression associations, GenAMap provides links to tools that allow the analyst to explore the discovered association. For example, consider a case where the analyst considers a discovered SNP-phenotypic trait association. The analyst can query dbSNP to find out information about the SNP, and the analyst can use GenAMap to visualize the frequency distribution of the phenotypic trait by genotype.

From this view, GenAMap provides other tools for the analyst that link to further details about the SNPs and associations. Directly from this view, the analyst can query for and link to the dbSNP [78] page of any SNP. For binary traits, the analyst can select a SNP and request to view the frequency table of the trait by genotype. For continuous traits, the analyst can compare the distributions of the trait by genotype (Figure 12).

## Discussion

In this paper, we described the development and usage of GenAMap, a visual analytics software platform for GWAS and eQTL studies. GenAMap is a suite of algorithmic tools that provide ready-to-use access to cutting edge machine learning research in GWAS and eQTL analysis. Not only have we built GenAMap to provide access to state-of-the art analytic methods, we have designed visualizations to enable analysts to explore the sea of data that results from these types of algorithms. By building on tried-and-tested visualization principles, we have developed visualization strategies that will enable analysts to explore association results from any analysis. Through multiple-coordinated views, we provide analysts with the ability to explore the structure in the genome, transcriptome, and phenome simultaneously, while considering associations between the data types. We provide instant access to online databases, GO annotations, and association strengths. These tools enable the analyst to explore the data in ways that would not be possible using command-line query tools.

Furthermore, we have shown that GenAMap enables biological discovery through an analysis on the NIH heterogeneous stock mice dataset. By using the sparse structured multivariate and multitask regression (S^2^M^2^R) algorithms provided in GenAMap for structured association mapping, we have not only uncovered SNP-phenotypic trait associations, but have identified specific genes that are associated with the eQTL hotspots and the clinical traits themselves. Indeed, using additional data and more sophisticated techniques allows us to understand the biological mechanisms behind SNP-phenotypic trait associations. Understanding the biological mechanisms behind SNP-phenotypic trait associations brings us one step closer to the prevention and treatment of complex diseases.

To combat the increasing complexity of genetics analysis, we believe that research must follow a pattern of collaboration and cooperation between disciplines, even those as vastly different as genetics, information visualization, and machine learning. We expect that GenAMap serves as an exemplary foray into this type of multi-disciplinary collaboration to build a suite of tools and visualizations based on cutting-edge machine learning technology. The problems facing geneticists today are a near perfect-fit for visualization and machine learning. As these fields come together with solid collaboration, the potential for discovery will continue to accelerate.

In the future, we plan to include other advanced structured association algorithms such as GroupSpAM [85] that allow for nonlinear genetic effects, as well as mStruct [86], Spectrum [87], and structured input-output lasso [30] that explore populational, genomic, and transcriptomic structures more comprehensively.

## Supporting Information

Figure S1 **Mouse gene network analysis.**
(PDF)

Figure S2 **eQTLs found in hippocampus tissue.**
(PDF)

Figure S3 **Gene expression-phenotypic trait associations for genes associated with chromosome 14.**
(PDF)

Figure S4 **Joint SNP-gene expression-phenotypic trait associations from chromosome 14.**
(PDF)

Figure S5 **Immunity associations from chromosome 17.**
(PDF)
