# Supplementary material for: GWAS in a Box: Statistical and Visual Analytics of Structured Associations via GenAMap
Source: PLoS One. 2014 Jun 6;9(6):e97524. doi: 10.1371/journal.pone.0097524 (PMC4048179; doi:10.1371/journal.pone.0097524)
Supplement: Figure S1 — Mouse gene network analysis. (PDF) [file pone.0097524.s001.pdf]

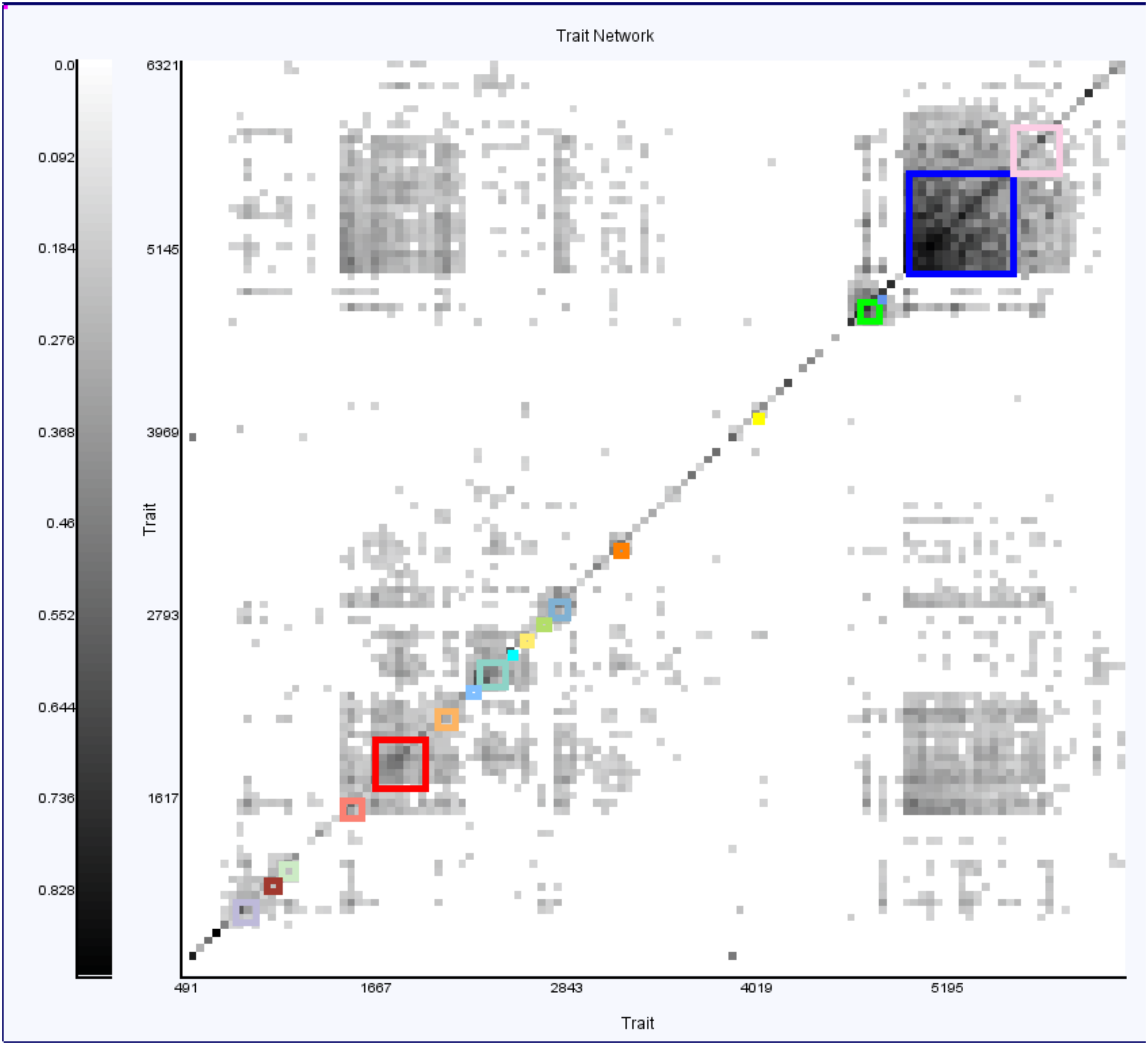

Figure S1. Mouse gene network analysis.
We used GenAMap to create gene-gene networks from the expression data for each tissue. GenAMap finds the top 20 connected modules and GO and eQTL enrichment for each module. Here, we show the gene-gene network generated using the hippocampus gene expression data.
