# Supplementary material for: GWAS in a Box: Statistical and Visual Analytics of Structured Associations via GenAMap
Source: PLoS One. 2014 Jun 6;9(6):e97524. doi: 10.1371/journal.pone.0097524 (PMC4048179; doi:10.1371/journal.pone.0097524)
Supplement: Figure S2 — eQTLs found in hippocampus tissue. (PDF) [file pone.0097524.s002.pdf]

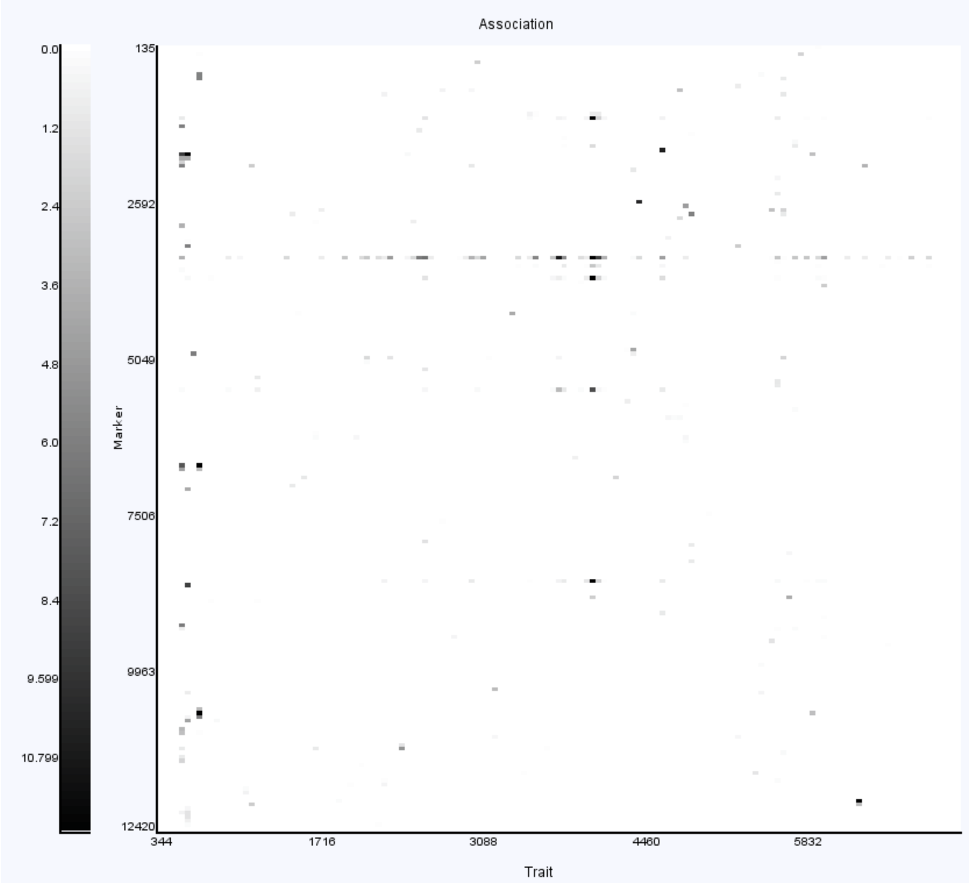

Figure S2. eQTLs found in hippocampus tissue.
We used GenAMap to find SNP-gene associations in the hippocampus gene expression data using GFlasso. In this figure, we show the overview of the results in GenAMap. This is a heat chart representation of the associations, where SNPs are represented along the y axis and the clustered genes are represented along the x axis. We have zoomed into the section of the gene graph where there are the most associations. We note an eQTL hotspot (represented by a horizontal line of associations), suggesting an eQTL hotspot that regulates many genes in trans.
