# Supplementary material for: GWAS in a Box: Statistical and Visual Analytics of Structured Associations via GenAMap
Source: PLoS One. 2014 Jun 6;9(6):e97524. doi: 10.1371/journal.pone.0097524 (PMC4048179; doi:10.1371/journal.pone.0097524)
Supplement: Figure S4 — Joint SNP-gene expression-phenotypic trait associations from chromosome 14. (PDF) [file pone.0097524.s004.pdf]

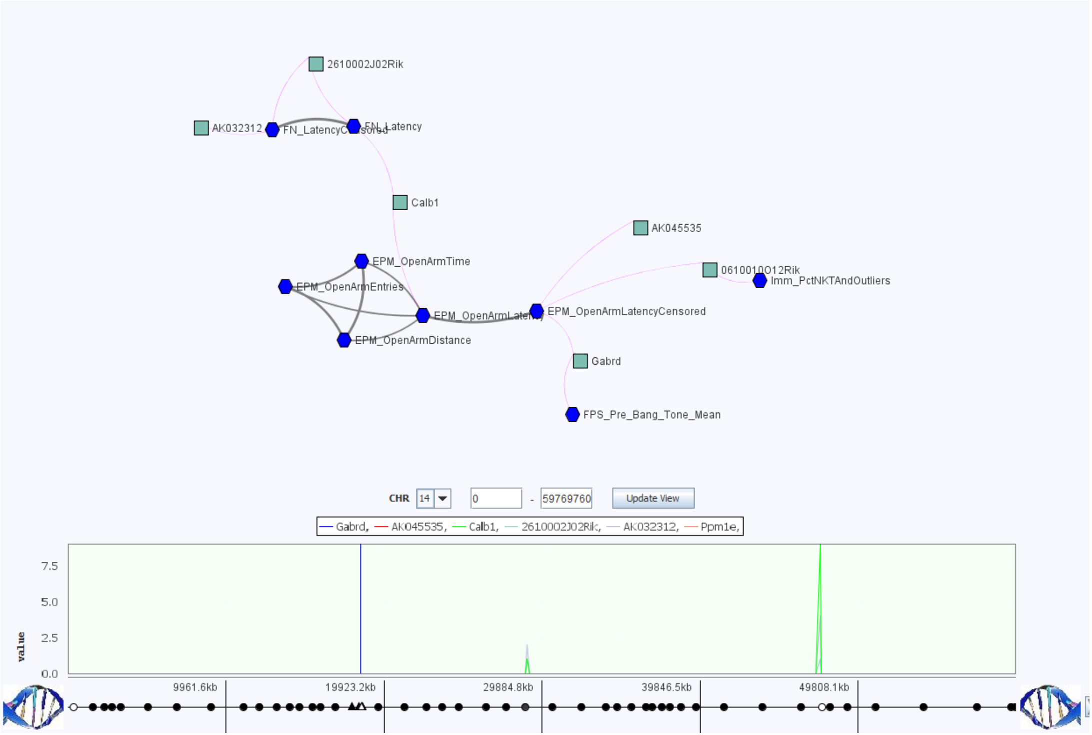

Figure S4. Joint SNP-gene expression-phenotypic trait associations from chromosome 14. We found a subnetwork of phenotypic traits and associated genes involved in brain function. The expression of these genes were also associated with the overlapping eQTL hotspots on chromosome 14.
