# Supplementary material for: GWAS in a Box: Statistical and Visual Analytics of Structured Associations via GenAMap
Source: PLoS One. 2014 Jun 6;9(6):e97524. doi: 10.1371/journal.pone.0097524 (PMC4048179; doi:10.1371/journal.pone.0097524)
Supplement: Figure S5 — Immunity associations from chromosome 17. (PDF) [file pone.0097524.s005.pdf]

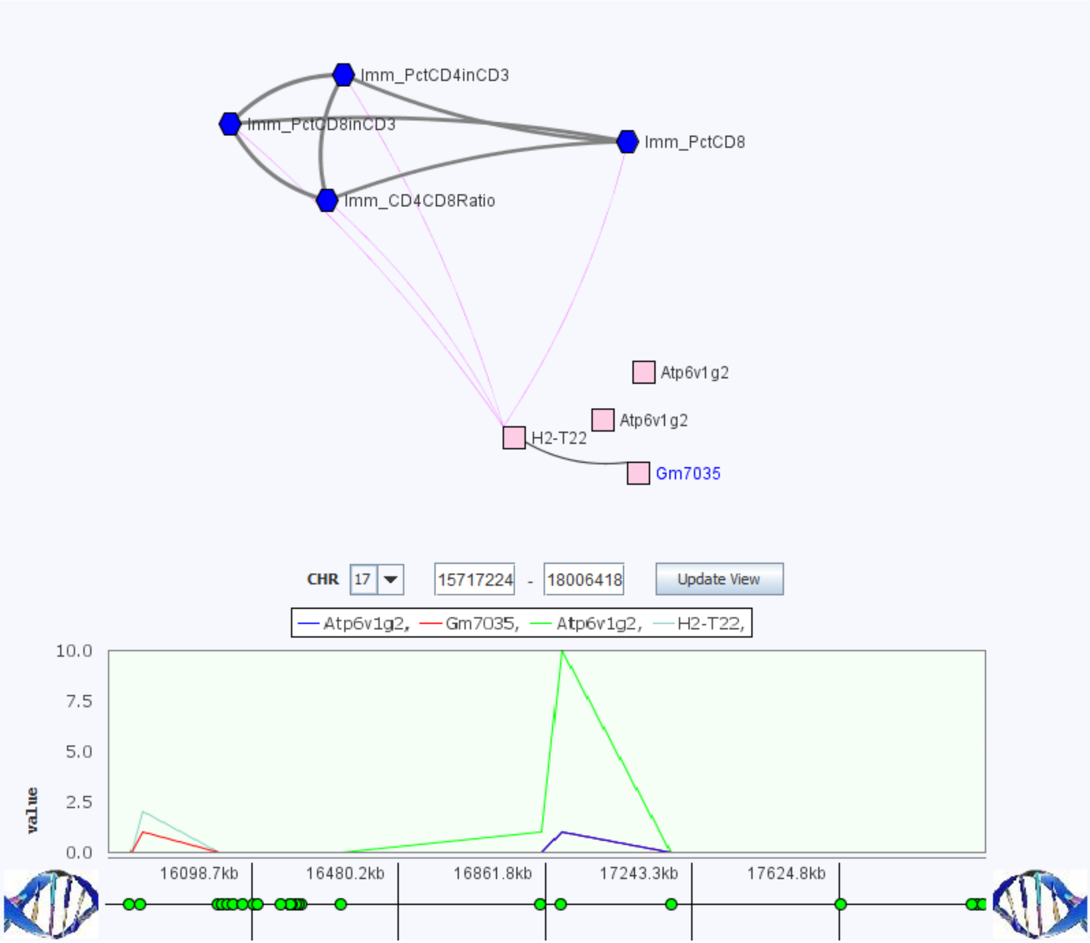

Figure S5. Immunity associations from chromosome 17.
We found a small group of genes associated with the H2 region on chromosome 17. We also found that these genes were associated with a subset of immunology traits.
