# Supplementary material for: GWAS in a Box: Statistical and Visual Analytics of Structured Associations via GenAMap
Source: PLoS One. 2014 Jun 6;9(6):e97524. doi: 10.1371/journal.pone.0097524 (PMC4048179; doi:10.1371/journal.pone.0097524)
Supplement: Figure S3 — Gene expression-phenotypic trait associations for genes associated with chromosome 14. (PDF) [file pone.0097524.s003.pdf]

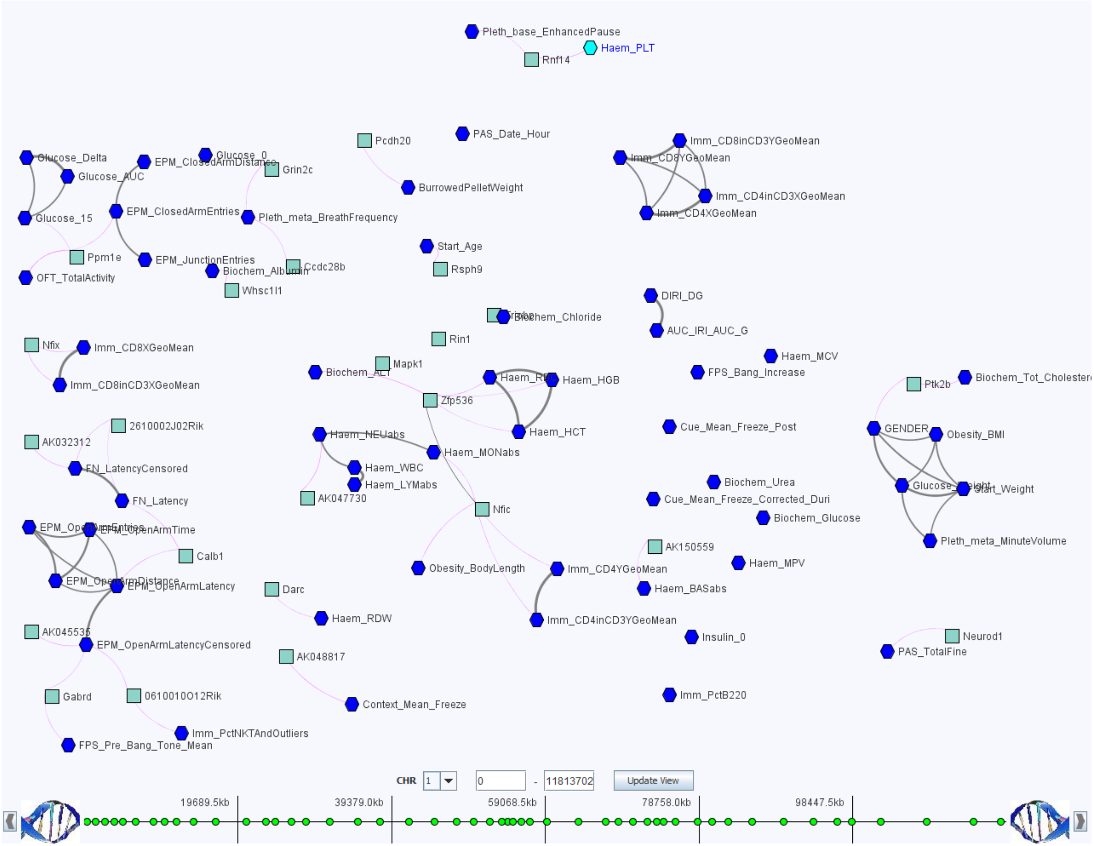

Figure S3. Gene expression-phenotypic trait associations for genes associated with chromosome 14.
We used GenAMap to explore the joint three-way associations corresponding to the eQTL hotspot on chromosome 14. We removed all other gene groups and then expanded the group to see the individual genes (squares). We filtered out all phenotypic traits that have no associations to the expression of these genes.
